# Optimizing Antibody Affinity and Developability Using a Framework–CDR Shuffling Approach—Application to an Anti-SARS-CoV-2 Antibody

**DOI:** 10.3390/v14122694

**Published:** 2022-11-30

**Authors:** Ranjani Gopal, Emmett Fitzpatrick, Niharika Pentakota, Akila Jayaraman, Kannan Tharakaraman, Ishan Capila

**Affiliations:** 1 Discovery and Diagnostics Division, Peritia Inc., 12 Gill Street, Woburn, MA 01801, USA; 2 Celltas Biosciences, 900 Middlesex Turnpike, Billerica, MA 01821, USA

**Keywords:** antibody, framework, CDR, affinity, SARS-CoV-2, COVID-19

## Abstract

The computational methods used for engineering antibodies for clinical development have undergone a transformation from three-dimensional structure-guided approaches to artificial-intelligence- and machine-learning-based approaches that leverage the large sequence data space of hundreds of millions of antibodies generated by next-generation sequencing (NGS) studies. Building on the wealth of available sequence data, we implemented a computational shuffling approach to antibody components, using the complementarity-determining region (CDR) and the framework region (FWR) to optimize an antibody for improved affinity and developability. This approach uses a set of rules to suitably combine the CDRs and FWRs derived from naturally occurring antibody sequences to engineer an antibody with high affinity and specificity. To illustrate this approach, we selected a representative SARS-CoV-2-neutralizing antibody, H4, which was identified and isolated previously based on the predominant germlines that were employed in a human host to target the SARS-CoV-2-human ACE2 receptor interaction. Compared to screening vast CDR libraries for affinity enhancements, our approach identified fewer than 100 antibody framework–CDR combinations, from which we screened and selected an antibody (CB79) that showed a reduced dissociation rate and improved affinity against the SARS-CoV-2 spike protein (7-fold) when compared to H4. The improved affinity also translated into improved neutralization (>75-fold improvement) of SARS-CoV-2. Our rapid and robust approach for optimizing antibodies from parts without the need for tedious structure-guided CDR optimization will have broad utility for biotechnological applications.

## 1. Introduction

The engineering of antibodies to develop them as therapeutics has shown tremendous success in treating a variety of disease conditions [1]. Importantly, the target-binding affinity and biophysical properties (yield, thermostability, solubility) are important critical quality attributes of therapeutic antibodies that impact their clinical development and the time needed to get them to market [2]. Affinity maturation, which is implemented at an early stage in the antibody development cycle, is achieved via two different methods: (1) a panning approach, whereby error-prone PCR or saturation mutagenesis is used to create libraries (to the order of 10^4^ or more designs) that are then screened via display methods [3]; (2) site-specific mutations (often guided by computational methods and three-dimensional structures or models) are used to generate a limited number of variants [4,5]. While both of these methods have successfully resulted in antibodies with substantially improved target-binding affinities, they are not amenable to optimizing multiple antibody quality attributes simultaneously, as mutations made to improve affinity may have a detrimental effect on other properties (e.g., yield or stability) [6]. The panning approach does not thoroughly sample the naturally occurring antibody diversity and runs the risk of introducing mutations not found in the natural antibody repertoire. The latter approach requires experimentally determined three-dimensional structures, which may not be available for new antibody–antigen interactions.

Over the past decade, we have been developing tools and methods for engineering antibodies to rapidly develop them as successful therapeutic candidates [7,8,9,10,11]. The principle behind our methods is to treat antibodies as distinct combinations of framework and CDR regions and capture key relationships between sequence, structure, and interaction network -based properties [11,12]. These properties are used to computationally screen the pre-existing vast knowledge base on the sequences and structures of antibodies and antibody–antigen complexes to generate a small grid of antibody constructs, which are then experimentally screened for target affinity and other developability properties.

Using our approach, we tackled the challenging problem of engineering an antibody that potently neutralizes the full spectrum of dengue virus serotypes (DENV-1 to DENV-4) using the template of a known antibody 4e11 that neutralized only serotypes DENV-1 to DENV-3 [5,8]. The resulting antibody Ab513 showed more than a 450-fold enhancement in binding to the DENV-4 serotype (compared to 4e11), showed highly potent in vitro and in vivo full-spectrum dengue virus neutralization, and showed a good safety and PK profile in phase 1 studies [5,8,12,13]. We also extended our approach to engineer a novel antibody ZAb_FLEP that specifically neutralizes the Zika virus, which showed good potency in vitro and in vivo [11] and showed successful clinical safety and efficacy. ZAb_FLEP was engineered by combining CDR loop features from other antibodies (including an antibody C8 that potently neutralized dengue viruses) and framework regions that comprise heavy and light chains from two different antibodies, thereby highlighting the utility and value of our antibody parts approach [11,14]. This parts-based approach to engineering antibodies has permitted us to rapidly generate and advance lead candidates optimized simultaneously for specificity and affinity to target epitopes and for developability by screening a significantly smaller set of variants compared to other panning and surface display methods.

The advances in next-generation sequencing together with isolating antibody-producing B-cells from patients have led to an explosion of sequence information on human antibodies, including germline information and heavy- and light-chain sequences [15]. The wealth of sequence information together with advances in machine learning algorithms have led to a transformative approach to antibody engineering [16,17,18,19,20]. Recent advances in next-generation sequencing technologies have produced high-quality sequence datasets that are expanding our insights on CDR usage and plasticity and antigen-driven evolution tracing (also known as affinity maturation). 

Taking advantage of the vast sequence information space of human antibodies, herein we implemented a computational CDR–FWR shuffling approach making use of pre-existing FWR and CDR fragments from the antibody sequences database to rapidly create new structures of the variable regions (Fv) and optimize a SARS-CoV-2 (template) antibody. We selected H4 [21] as the template based on the prevalence of its germline in the natural immunogenic response to SARS-CoV-2 infection in convalescing patients. Antibody H4 binds the receptor-binding domain (RBD) of SARS-CoV-2 with high affinity and neutralizes the virus by blocking the interaction between the RBD and the host’s receptor (ACE2) [21]. The shuffling process involves searching an antibody sequence database for framework and CDR regions to assemble functional antibodies with optimized affinity and developability. The sequence database was compiled by collecting data published in a small number of studies that performed next-generation sequencing of Ig repertoires from infected or vaccinated individuals. The CDR sampling strategy follows three steps: (1) predicting the CDR sites (‘paratope’) that are likely to be involved in interacting with the target epitope [22]; (2) mining the antibody sequences database to identify CDR homologs having mutations at one or more paratope sites; (3) ranking the homologs based on their frequency of occurrence in the database and the physiochemical differences between the AA mutations in these homologs relative to the template CDRs. In the FWR sampling strategy, frameworks that satisfy (1) the germline usage in the antibody repertoire from COVID-19 convalescent patients, (2) the number of CD4+ T-cell epitopes, (3) the sequence diversity from the germline (viz. somatic hypermutations), and other (4) in silico developability guidelines established for therapeutic antibodies [23] are selected from the database. Then, the pre-selected CDR homologs are combined with the FWRs using an interatomic interactions network to form a focused library of antibody designs. Through the CDR sampling strategy employed in this work, we generated a candidate CB79 with >5-fold enhanced affinity (compared to H4 antibody) by experimentally screening less than 100 computationally designed variants. This affinity enhancement is on par with other efforts using evolution techniques that screen hundred thousand to millions of variants [24]. The lead candidate, CB79, also exhibits improved in vitro neutralization (>75-fold) and in silico developability properties relative to H4. 

## 2. Results 

**Figure 1:**
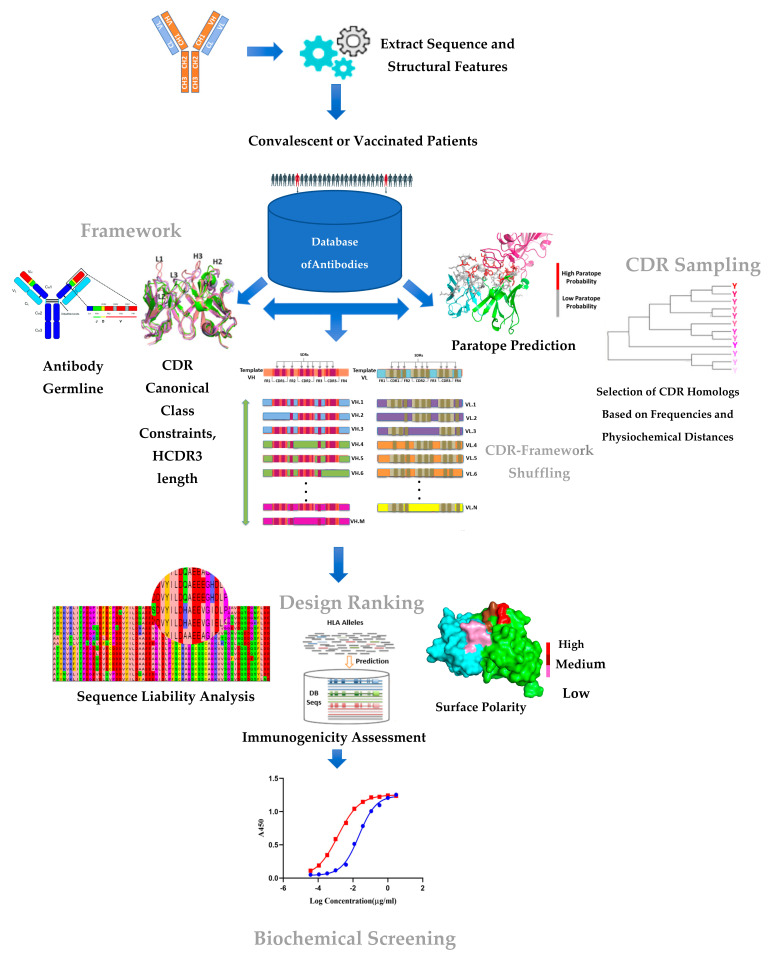
Key components of the CDR–FWR shuffling approach. Starting from a template antibody (selected based on its potency and mechanism of action), the approach involves searching and screening (referred to as sampling) for CDR parts and FWR parts from a large sequence repository of human antibody sequences. The metrics used in the sampling FWR (germline properties, HCDR3 length, etc.) and CDR (paratope prediction and amino acid substitution based on natural frequencies, etc.) are shown. The design space generated by combining the sample CDRs and FWRs are assessed experimentally in a high-throughput manner for quality (expression, yield, thermal stability) and affinity (spot check binding to RBD antigen) to identify the lead candidate.

The main components of our parts-based approach to engineering antibodies (Figure 1) include a computational design element and experimental screening to assess the quality (such as expression and yield) and affinity (antigen-binding) of the designed antibody candidates. These components and their application to SARS-CoV-2 antibody engineering are described in the following.

### 2.1. A CDR–Framework Shuffling Approach Optimizing the Target-Binding Affinity and Developability

First, we sought to generate a CDR library with mutations that sample natural diversity while retaining specificity and stability. Several factors went into the design of the CDR library: (1) we restricted the CDR library to a manageable size to avoid combinatorial explosion and to keep the overall number of designs to less than a hundred; (2) to identify CDR homologs from the sequence database, which has over 4 million VH and VL sequences, we narrowed the search using canonical class constraints (for H3, we only considered those having a matching length to the H3 loop of H4); (3) we restricted the number of CDR mutations per loop to two; (4) we retained CDR loops having mutations at sites predicted to be involved in antigen recognition as predicted by the Parapred software program [22] and excluded the rest; (5) after a final filtering step to remove redundant entries, we scored the remaining loops based on the physicochemical properties of the substituted amino acids with respect to the H4 CDR sequence, with the rationale that amino acids with distinct physicochemical properties are more likely show changes in the binding properties (Figure 1). This search process identified CDR loops from 23 different CDR variants (L1: 4, L2: 2, L3: 3, H1: 5, H2: 3, H3: 6) (Table 1). 

We selected antibody frameworks based on their (1) germline usage in the convalescent antibody repertoire and (2) predicted developability properties. An analysis of the immunoglobulin repertoire from infected or convalescent individuals using next-generation sequencing showed that certain germlines (e.g., VH1-69, VH3-30) are preferentially used in targeting viral structural proteins in infectious pathogens such as HIV-1, RSV, dengue, and hepatitis C [25]. Therefore, an important component of our framework selection approach involved analyzing antibodies isolated from the B-cells of SARS-CoV-2 convalescent individuals to identify enriched V-J segments in heavy and light chains.

**Figure 2:**
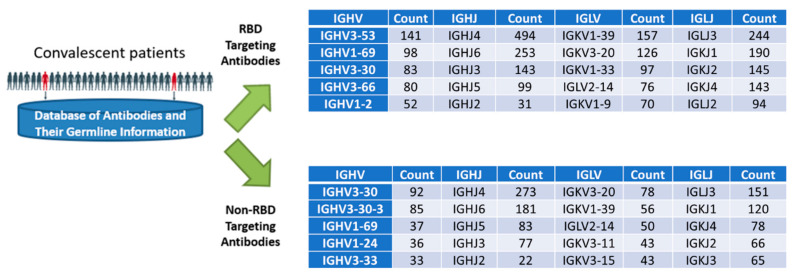
The distribution of heavy- and light-chain variable gene usage in 1145 RBD and 668 non-RBD antibodies isolated from convalescent patients. Antibody sequence and germline data were downloaded from CoV-AbDab [26].

From our sequence analysis, we observed a heterogenous pattern of heavy- and light-chain variable gene usage in antibodies isolated from convalescent patients (Figure 2). Importantly, distinct differences were seen in the gene usage of RBD-targeting antibodies versus non-RBD-targeting antibodies. Figure 2 shows the top 5 enriched V and J genes in a total of 1145 RBD and 668 non-RBD antibodies collected from the public repository CoV-AbDab [26]. For instance, IGHV3-53 and IGHV1-2, which are enriched in RBD-targeting antibodies, are not enriched in non-RBD antibodies; similarly, IGHV1-24 was found to be enriched in the non-RBD antibodies but not in the RBD antibody population. Our framework selection process assigned higher weights to candidates that belonged to enriched germlines, especially VL: IGKV2-40, JK4, VH: IGHV1-2, JH2, since it also happens to encode antibody H4 (in practice, the template antibody may not always belong to an enriched class of germline), and excluded candidates that did not belong to the enriched gene families (i.e., not in the top 5 enriched genes).

**Table 1:** CDR library design for antibody affinity maturation. The CDR sites selected for mutations were identified using the scores (‘Parapred score’) assigned by the antibody paratope prediction software, Parapred. Residue substitutions within each CDR were selected based on a combination of factors: (1) the frequencies of amino acids within a canonical class (shown as percent fraction values within the cells); (2) the most commonly occurring CDR homologs within the sequence database (last column of the table); (3) the physiochemical distance between the wild-type residue and the mutant. The CDR homologs are assigned into different groups (2nd column) and assigned distinct labels (also used in Figure 3 and Table 2).

**Loop**	**Group**	**23**	**24**	**25**	**26**	**27**	**28**	**29**	**30**	**31**	**32**	**33**	**34**	**35**						**Full Loop DB Count**
H1	0	K,53.67	A,75.95	S,97.66	G,97.55	Y,42.87	T,70.22	F,91.99	T,37.38	G,6.32	Y,77.92	Y,15.09	M,46.29	H,30.46						27772
	1					F,39.16														20326
	2								S,43.75											1285
	3											W,21.37								220
	4									S,48.58										105
	5									D,11.18										3
Parapred Score		0	0	3.6	9.7	24	46	8.4	80	90	81	97	1.7	0						
**Loop**	**Group**	**50**	**51**	**52**	**52A**	**53**	**54**	**55**	**56**	**57**	**58**									**Full Loop DB Count**
H2	0	R,10.87	I,85.21	N,28.33	P,74.93	N,16.16	S,17.33	G,66.48	G,9.21	T,70.29	N,41.62									6726
	1						T,4.95													324
	2					S,10.18														70
	3			S,15.98																59
Parapred Score		84	3.6	93	45	91	81	23	72	45	63									
**Loop**	**Group**	**93**	**94**	**95**	**96**	**97**	**98**	**99**	**100**	**100A**	**100B**	**100C**	**100D**	**100E**	**100F**	**100G**	**100H**	**100I**	**101**	**102**
H3	0	A,92	R,76	V,7	P,7	Y,50	C,100	S,62	S,32	T,31	S,52	C,100	H,4	R,7	D,4	W,5	Y,18	F,70	D,80	L,7
	1																W,0.29			
	2																			I,14
	3														E,12					
	4										T,11									
	5												Y,64							
	6												Y,64	S,15						
Parapred Score		1.5	37	65	69	93	90	81	75	80	75	69	80	82	57	85	20	3.8	19	4.5
**Loop**	**Group**	**24**	**25**	**26**	**27**	**28**	**29**	**30**	**30A**	**30B**	**30C**	**30D**	**30E**	**30F**	**31**	**32**	**33**	**34**		**Full Loop DB Count**
L1	0	R,7.74	S,97.64	S,97.50	Q,94.51	S,85.60	L,13.94	L,88.90	D,4.35	S,74.50	D,2.57	D,7.77	G,3.49	N,5.35	T,3.47	Y,87.81	L,96.02	D,3.13		695
	1	K,90.02																		4
	2						I,10.89													1
	3														N,88.62					0
	4												S,3.57							0
Parapred Score		0.9	0.2	1.4	18	13	5.6	23	76	63	79	78	50	68	6.3	85	0.1	14		
**Loop**	**Group**	**49**	**50**	**51**	**52**	**53**	**54**	**55**	**56**											**Full Loop DB Count**
L2	0	Y,87.77	T,1.44	L,0.25	S,74.94	Y,1.21	R,71.09	A,26.76	S,69.71											774
	1		S,4.28																	33
	2			V,15.37																32
Parapred Score		57	66	5.8	31	81	20	29	45											
**Loop**	**Group**	**89**	**90**	**91**	**92**	**93**	**94**	**95**	**96**	**97**										**Full Loop DB Count**
L3	0	M,11.33	Q,91.15	R,7.35	I,1.79	E,1.20	F,7.13	P,85.24	L,20.64	T,88.31										56
	1						W,16.28													34
	2								I,5.94											20
	3				L,8.20															0
Parapred Score		8.7	4.5	92	91	92	88	11	45	0.9										

**Table 2:** Antibody frameworks selected for the shuffling process. For each framework, the internal accession id (CAB_NO) and group classification are provided.

VH Framework			VL Framework		
HF_Group	Scaffold (Accession No.)	Sequence	LF_Group	Scaffold (Accession No.)	Sequence
1	CAB_NO: 1036253	EVQLVQSGAEVKKPGASVKVACKASGYTFTDYYIHWVRQAPGQGLEWMGRINPNSGGTNYAQNFQGRVTMTRDTSINTASMELSRLTSDDTAVYYCARRGYCSGGSCYGGDYFDYWGQGTLVTVSS	1	CAB_NO:319636	DIVLTQSPLSLPVTPGETASISCTSSQSLLDRDDGNTYLDWYLQKPGQSPQLLIYTLSSRASGVPDRFSASGSGTDFTLKISGVEAEDVGVYYCMQRIEFPLTFGGGTKLEIK
2	CAB_NO:33048	QVQLVQSGAEVKKPGSSVKVSCKASGGTFSSYAISWVRQAPGQGLEWMGGIIPIFGTANYAQKFQGRVTITADKSTSTAYMELSSLRSEDTAVYYCAREVDCSSTSCYGSWYFDLWGRGTLVTVSS	2	CAB_NO: 355327	DIVLTQTPLSLPVTPGEPASMSCRSSQSLLDSDDGNTYLDWYLQKPGQSPQLLIHTLSYRASGVSDRFSGSGSGTDFTLKISRVEADDVGVYYCMQRTQFPLTFGGGTKVEIK
3	CAB_NO:1142140	QVQLVESGAEVKRPGASVKVSCKASGYTFTSSPIHWVRQAPGQGLQWMGLINPGGGTSTFAQRFQGRVTMTRDTSTNTVYMDLSGLRSEDTAMYYCARAPSYDSGGSFPADYLDYWGQGTLVTVSS	3	CAB_NO:11070	DVLMTQTPLSLPVSLGDQASISCRSSQSLVHSDGNTYLEWYLQKPGQSPNLLIYKLSNRFSGVPDRFSGSGSGTDFTLKISRVEAEDLGVYYCFQGSHVPPTFGGGTKLEIK
4	CAB_NO:454656	QVHLVQSGAEVKKPGASVKVSCKASGYTFTSHGISWVRQAPGQGLEWMGWISTYNGNTNYPETLQGRVTMTTDTSTSTAYLEVRSLTPDDTAVYYCARVGCRSTSCWAGTHWFDPWGQGTLVTVSS			

**Figure 3:**
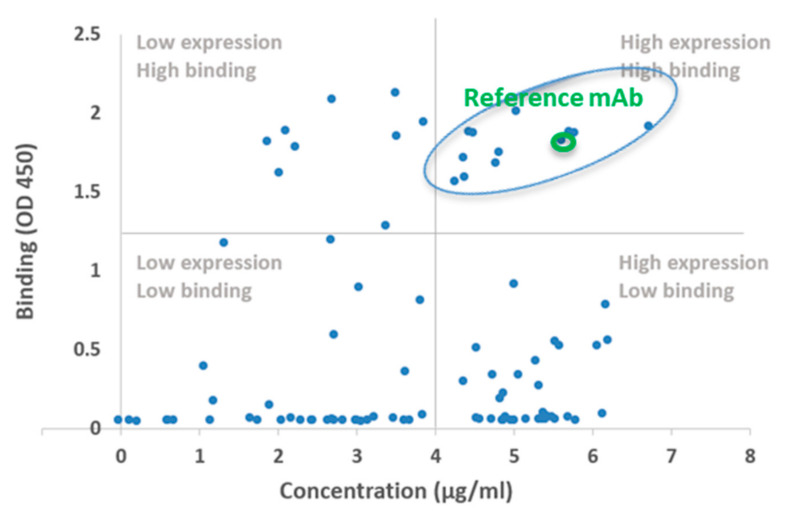
The genesis of CB79 using a CDR–FWR shuffling approach. The X-axis shows the protein expression level as protein concentration obtained from 1 mL and the Y-axis shows the OD value from the ELISA-based screening of antigen binding (see Methods) in the designed antibodies. CB79 was selected from candidates in the quadrant of high expression and high binding based on its improved properties, including its in silico developability and binding compared to H4. The nomenclature of the designed constructs and their heavy- and light-chain groups (from Table 1 and Table 2) and their expression and antigen-binding properties relative to the template H4 antibody are shown in Supplementary Figure S2. Antibodies that expressed and showed RBD binding comparable to (or greater than) H4 (highlighted by the green circle) and satisfied the immunogenicity and developability criteria were identified and taken forward for additional screening for ACE2 inhibition and in vitro neutralization.

Then, the potential VH and VL frameworks were matched with the pre-selected CDR homologs to determine the feasibility of combining them to form functional Fv fragments. The matching process considered only those antibody frameworks that possess similar CDR canonical classes and VH/VL family subgroups as those of the donor antibodies (Table 2). Creating a shuffled variable antibody domain with distinct VH and VL FWRs and various CDR regions can lead to non-functional antibodies due to incompatibility between the selected CDRs and the FWR regions. To avoid this, we examined and optimized the network of interactions at critical interfaces between heavy and light chains and between CDRs and FWRs by borrowing structure-determining residues (or SDRs) from H4 (Figure 1). The compatibility between a pair of antibody frameworks (heavy and light) and CDR homologs was evaluated computationally as follows. First, to maintain and stabilize the three-dimensional conformation of the CDR loops to provide optimal contact with the epitope surface, it is important to ensure that the network of contacts between the proximal framework residues and CDR loops and the VH–VL interface are optimized. This implies having amino acids from the template antibody at appropriate framework positions to support the different interfaces. The scaffold selection process attempts to select VH and VL frameworks that have residues matching the template antibody in these framework positions, thereby minimizing any additional structural engineering. Back mutations are introduced into the frameworks in cases of residue mismatches in these critical residue positions. Finally, 3D models of the resulting VH and VL structures are built and analyzed for potential steric clashes that destabilize the interface. Back mutations from H4 are introduced, if necessary, to minimize unfavorable stearic contacts. This process led to the selection of seven VH/VL framework regions (4 VH and 3 VL) from diverse antibodies (Table 2), which were then used to generate an initial set of designs (Figure 1).

Previous studies have shown that antibodies that are closer to the germline (i.e., possess fewer somatic hypermutations) are associated with increased levels of polyreactivity [27]. It was shown that mAbs with >20 amino acid substitutions from the germline had a lowest propensity (1%) to cause polyreactivity [27]. Our method introduces multiple mutations into conserved framework regions, as well as the CDRs of both heavy and light chains; therefore, our designed candidates possessed on average 15-20 mutations in VH and VL. Finally, to minimize the risk that these mutations might generate new T-cell epitopes, which could cause anti-drug antibody (ADA) responses in humans, we evaluated the designed VH and VL candidates using the netMHCIIPan3.2 software, which predicts the binding sites of MHC class II molecules [28]. Finally, the candidate VH and VL were screened using the in silico developability prediction software TAP (Therapeutic Antibody Profiler [23]), which uses key criteria such as the CDR length and surface polarity of the molecule to evaluate the developability profile of the candidate. Framework–CDR combinations ranked highly by the immunogenicity and developability metrics were taken forward for the biochemical analysis (Figure 1C). The overall design process led to a total of 12 VHs and 7 VLs, each having between 1-3 CDR mutations.

### 2.2. Biochemical and In Vitro Characterization of SARS-CoV-2 Antibodies

**Figure 4:**
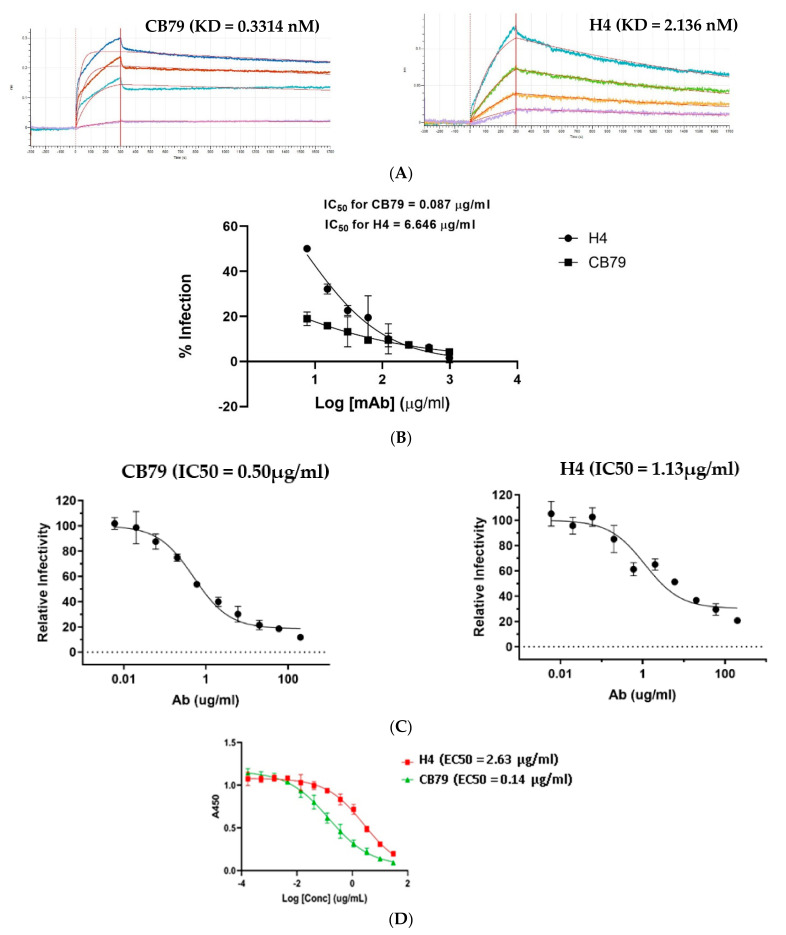
Biochemical and in vitro potency of CB79 compared to that of H4: (**A**) the binding kinetics of CB79 and H4 measured using biolayer interferometry on the Octet instrument; (**B**) PRNT-based neutralization using a live SARS-CoV-2 virus of CB79 and H4 as a function of the reduction in infection upon antibody treatment; (**C**) the neutralization of the pseudovirus comprising the SARS-CoV-2 spike protein of CB79 (left panel) compared to H4 (right panel); (**D**) the relative potency of CB79 and H4 in inhibiting the binding of the SARS-CoV-2 spike protein RBD to the human ACE2 receptor.

The designed heavy and light chains were paired against each other and the resulting grid of 84 combinations were recombinantly expressed as human IgG1 and screened for binding to the RBD and S1 subunit (which comprises the N-terminal domain and RBD) of SARS-CoV-2 using a spot ELISA (Methods). Several of the designed constructs expressed poorly and/or bound at a substantially lower affinity when compared to H4, although some of these constructs had only 2 mutations within the CDRs (Figure 3A,B). Notably, these poorly performing constructs had distinct frameworks compared to the reference antibody, supporting the importance of CDR–FWR compatibility to preserve the folding and binding properties [14,29] (Figure 3A,B). The binding properties of our designed mAbs clearly pointed out that the engagement of the mAb with the epitope to achieve a threshold binding affinity critically depends on the CDR and the framework in the context of the contacts they make with the epitope and the inter-residue interaction network between the framework and CDR. Roughly one-fifth of the designs showed high binding to RBD (comparable to or better than the reference RBD antibody) (Figure 3A). Based on its expression, binding, and predicted developability attributes, we chose CB79 as the lead candidate. As expected, the immunogenicity scores of CB79 were within the range predicted for the clinical-stage therapeutic (CST) antibodies (human or humanized mAbs in trials or approved as of November 2019 (Supplementary Figure S1). Typically, the in silico demonstration of low immunogenicity risks correlates with minimal toxicity effects, including anti-drug antibodies, in non-clinical toxicology and clinical studies. Some of the designed candidates exhibited higher binding signals but they expressed these at relatively lower levels (Supplementary Figure S2). The purity and thermal stability of CB79 was confirmed using SEC and DSC, respectively (Supplementary Figure S3). Next, we investigated the impacts of the distinct framework–CDR relationships of our designed candidates on the kinetics of the binding, such as the on-rate and off-rate to their epitope on the RBD. The analysis of the binding kinetics of CB79 to the RBD using biolayer interferometry showed a >6-fold improvement in binding to the RBD (0.33 nM) compared to H4 (Figure 4A).

The predicted slower off-rate of CB79 would enable the antibody to be engaged to the antigen for a longer duration, leading to an improvement in the neutralization of SARS-CoV-2. Subsequently, we tested CB79 for the neutralization of live SARS-CoV-2 in a PRNT assay (Methods). Consistent with our prediction, CB79 showed >75-fold improved neutralizing potency towards SARS-CoV-2 compared to H4 (Figure 4B). The superior neutralization of CB79 over H4 was also observed in a pseudovirus neutralization assay (Methods (Figure 4C). To verify whether the mutations in CB79 impacted its ability to bind to the spike protein and block its binding to the ACE2 receptor, we evaluated the ACE2-blocking potential of CB79 using a biochemical assay (Methods). Compared to H4, CB79 showed >18-fold improved ACE2 blocking potential (Figure 4D). The data also demonstrated that the mechanism of action of CB79 to neutralize SARS-CoV-2 paralleled the neutralization of the virus by H4, and that the increased neutralization potency is a result of the slower off-rate.

## 3. Discussion

Extending our journey in antibody engineering using known sequence and structural data in public databases, we developed and demonstrated a CDR–FWR shuffling approach that uses extensive data from antibody sequence databases to engineer new antibodies. Our approach does not rely on structural data for the target epitope, given that the CDR library is generated by picking the most frequently occurring CDR homologs from the natural antibody sequences database, without guidance from the target epitope. The key to the success of our approach relies on taking advantage of the pre-existing knowledge of human antibodies (which are naturally optimized for safety and efficacy in humans) to synthesize new variants with an increased likelihood of generating successful lead candidates. Furthermore, our methodology restricts the sequence space to naturally occurring Ig antibodies, minimizing the risk of introducing any undesired T-cell epitopes. When compared to a typical structure-guided CDR redesign process that involves making mutations to improve contacts with specific residues on the target epitope, our CDR–FWR shuffling process described here relies on diversifying CDRs through minimalistic changes using a largely structure-independent fashion. Therefore, we were able to affinity mature an antibody by sampling a limited search space (n < 100), without compromising on thermal stability or other biophysical properties. Consistent with the optimization of the developability, our lead SARS-CoV-2 antibody showed good expression in terms of the chemistry manufacturing controls (CMC) during its production under Good Manufacturing Practice (GMP) conditions and no adverse effects in the non-clinical toxicology and phase I safety evaluation in humans. Its pharmacokinetics properties in phase I human studies were on par with other clinical stage antibodies belonging to the same isotype.

To determine whether the published cryo-EM structure of the H4–spike protein complex [30] could have guided the CDR mutations instead of the CDR sampling strategy, we compared the sequences and structural models of our engineered antibody CB79 with that of H4 (Supplementary Figures S4 and S5). Only three mutations (G31D mutations of HCDR1 and H100DY, R100ES mutation of HCDR3) were in proximity to the epitope surface, and among them only G31 seemed to be at a distance to contact the antigen, whereas the side chains of H100D and R100E were facing away from the epitope and were at least 4 A° from the nearest RBD residue (Supplementary Figure S4). To understand the impact of these mutations, we performed two sets of parallel experiments. In one experiment we introduced single mutations on H4 to determine whether one or more of these would improve the binding to RBD of the spike protein, and in the other experiment we introduced reverse mutations on CB79 to determine whether one or more of these back mutations would reduce the binding signal closer to H4. The forward and reverse mutations respectively on H4 and CB79 did not show significant impacts on the binding affinity to the spike protein RBD (Supplementary Table S1). Therefore, these mutations would not be selected by structure-guided approaches that aim to improve contacts based on the cryo-EM of H4–RBD complex. Additionally, the advantage of our CDR sampling approach is highlighted by the value in combining various CDR parts (HCDR1 and HCDR3) comprising these mutations to significantly improve the affinity of the engineered antibody CB79 over that of H4 for the spike protein RBD. Specifically, the unintentional inclusion of the (low) affinity-enhancing variants within a limited CDR library of 23 variants highlights the potential value in scaling this process (i.e., sampling a larger library of CDRs). As such, the principles employed in CDR sampling and selection are similar to those employed in directed evolution methods reported elsewhere [24].

With the availability of a wealth of human antibody sequence information, other strategies to diversify CDRs and improve their binding affinity using various machine learning models have been developed. In one study, a strategy for CDR diversification resulted in improving the affinity of the anti-α-synuclein nanobody by 4.5 times [24]. In another study, large language models (LLMs) were employed to improve the antibody affinity without using epitope information, which showed promising initial experimental results against a variety of infectious disease targets [31]. Specifically, 6 protein (not antibody specific) LLMs developed by Facebook [32] were used to assess potential mutations by calculating the likelihood that a given amino acid would occur at a specific position in the context of the rest of the sequence. Both the LLM method and our CDR–FWR shuffling approach described herein rely on the hypothesis that sampling a small number of constructs using patterns in antibody sequence databases will yield functionally different mAbs. Importantly, these recent methods including our approach highlight the importance of non-CDR mutations in modulating the function and developability of the antibody. Our approach also complements the other LLM and machine learning approaches in that the entire VH and VL frameworks are diversified and the structural knowledge of the paratope is also factored in to select appropriate CDRs in the shuffling process.

With advances in machine learning methods and high-throughput screening technologies, our methodology has significant potential to grow [33]. Recent evidence indicates that framework residues may positively impact antigen binding through a variety of mechanisms, including long-range interactions, the stabilization of CDR backbones, the reorientation of the heavy- and light-chain interface, and surface–solvent interactions [29]. Thus, sampling more antibody frameworks as part of the CDR–FWR shuffling process could increase the chances of finding candidates having favorable antigen-binding properties relative to the template. Additionally, increasing the CDR sample space, sampling paratope features from two or more templates (in the case of H4 optimization, these would include mAbs that target class 1 epitope), capturing co-evolving antibody residues, implementing feedback between designs, and conducting experiments to improve the success rate are important avenues to further improve our methodology. There are many templates of antibodies that target epitope surfaces on the spike protein that are conserved across SARS-CoV-2 variants and multiple coronaviruses, although these antibodies may not be equally potent across the different viruses and the variants. Our CDR–FWR shuffling approach can be used to engineer antibodies by building on these templates, which would show potent neutralization across different coronaviruses. Finally, combining our approach with recently published machine-learning-based approaches is a direction for future research in this exciting era of machine-learning-guided antibody engineering.

## 4. Methods

### 4.1. Databases Employed

COVID-19 Sequence Database (germline analysis): Whole Fv sequences belonging to SARS-CoV-2–specific antibodies isolated from COVID-19 patients were retrieved from CoVAbDab [26] on 23 June 2020. The antibodies were grouped into two classes based on their target epitope—RBD or non-RBD—and the germline distribution of each group was plotted in the form of pie charts. The frequency of occurrence of a germline gene (V-D-J in the case of VH and V-J in the case of VL) was used as a surrogate measure of enrichment.

Antibody Sequence Database (CDR analysis and CDR–framework shuffling): The antibody database is a curated database consisting of filtered sequences from the cAb-rep IGG-Seq database [34] and SAbDAb structural DB database [35]. Sequences were included in the database if the ANARCI [36] assigned species was human and the number of AA deletions was less than 5, where an AA deletion is the absence of an amino acid at one of the non-insertion positions in the Chothia numbering scheme. All sequences were numbered using ANARCI. As of the time of this writing, there are 1,454,433 unique VH and 807,349 unique VL sequences in the database. During the pre-processing of the sequences, several attributes that can be used in downstream tasks are associated with each sequence. These include SCALOP [37]-assigned north CDR clusters, Chothia canonical classes [38], Chothia definition CDR loop lengths, and ANARCI-assigned germlines. This database was the basis for both the framework and CDR analyses that were used to engineer the antibodies in this paper.

### 4.2. Selection of Candidate CDRs for the Sampling Process

The CDR filtering that resulted in the final candidate CDRs proceeded as follows:

1. CDR filtering was used based on the north cluster and length to establish a set of candidate CDRs:
  - H1: length = 7, cluster = 13-A − 1154116 CDRs;
  - H2: length = 6, cluster = 10-A − 691070 CDRsH3: length = 17, cystine at position 98,100C – 2018 CDRs;
  - L1: length = 17, cluster = 16,17-A − 52446 CDRs;
  - L2: length = 7, cluster = 8-A – 793224 CDRs;
  - L3:length = 9,cluster = 9,10-A − 329011 CDRs.
2. Mutations present in the candidate set of CDRs were scored using the following metrics:
  - Frequency score: Here, $\log_{2}p\left({\mathrm{CDR}}_{\mathrm{AA},i}\right)-\log_{2}p({\mathrm{TEMPLATE}}_{\mathrm{AA},i})$, where $p\left({\mathrm{CDR}}_{\mathrm{AA},i}\right)$ is the probability of finding ${\mathrm{CDR}}_{\mathrm{AA}}$ at position i in the candidate set, and $p({\mathrm{TEMPLATE}}_{\mathrm{AA},i})$ is the probability of finding the template AA at position i in the candidate set. This score is meant to optimize the likelihood that the amino acid ${\mathrm{CDR}}_{\mathrm{AA},i}$ will be compatible with the structural properties of the paratope;
  - Paratope probability: The probability that the position in question is in the paratope, given the template CDR sequence, as calculated by the Parapred tool [22]. This score was used to try and mutate amino acids mainly in the epitope–paratope interface;
  - Blosum score: The Blosum62 matrix score for the AA substitution ${\mathrm{TEMPLATE}}_{\mathrm{AA}}$→ ${\mathrm{CDR}}_{\mathrm{AA}}$. This score was intended to optimize the likelihood that ${\mathrm{CDR}}_{\mathrm{AA},i}$ will be compatible with the structural properties of the paratope;
  - Substitution type: The type of AA substitution, where an identical AA = 0, substitution with an amino acid of the same class = 1, and substitution with an amino acid from a different class = 2. This score was intended to optimize the potential physiochemical changes in the epitope–paratope interface;
3. CDR candidate selection using metrics and database occurrence. Given the metrics defined above and the database occurrence statistics, CDRs from the candidate were selected or new CDRs were engineered using mutations from the candidate set (Table 1).

### 4.3. Selection of Candidate VH/VL Frameworks for CDR/FWR Shuffling

The frameworks were filtered in 4 stages. The initial stage identified candidate frameworks using coarse-grained filters. Subsequently, in stage 2, the template CDRs (without the CDR point mutations) were ported onto the candidate scaffolds and simple sequence-based metrics were computed. The template CDRs were used as a surrogate for the final engineered CDRs to avoid an exponential increase in designs that had to be filtered in silico. In stage 3, these scaffolds were sampled to maximize the diversity that was tested experimentally by sorting the scaffolds according to the calculated metrics and then sampling them, such that a minimum diversity in the final set was maintained. Finally, this set of scaffolds was scored for T-cell epitopes using netMHCIIpan3.2, and scaffolds where the fraction of alleles that contained a T-cell epitope at any position less than the 95th percentile of clinical stage antibodies were considered for final filtering.

Heavy-Chain Filtering Process

Stage 1:

1. North cluster: H1 = H1-13-A, H2 = H2-10-A, H3 length = 17:16,425 antibodies remaining;
2. Human germline identity > = 0.80: 16,231 antibodies remaining;
3. Orientation residues (36, 38, 42, 43, 44, 87, 98) match template residues (100% identity): 14,383;
4. Number of rare amino acids in the template (occurring in <1% of human residues in the antibody database) < = 10:9169.

Stage 2:

5. Total rare amino acids after template CDR porting. Given the template CDRs may have unusual residues, the scaffolds are re-filtered to have less than 5 rare amino acids after porting the template CDRs (note that this looks redundant compared to step 4, but is necessary because the database only stores the total number of rare amino acids);
6. The PKA score is computed as a simple heuristic for antibody charge.

Stage 3:

7. Scaffolds are sorted by their loop homology to the template, rare amino acids, and PKA scores and then sampled such that the final scaffold set will have a hamming distance of at least 12 between all final scaffolds; 40 scaffolds remained in the end.

Stage 4:

8. Scaffolds satisfying the T-cell epitope criterion (no position should have more than the maximum number of alleles flagged at that position among CSTs, and no position should have more than the 90th percentile of alleles flagged at that position among CSTs) are considered for use in the candidate designs; 8 scaffolds remained in the end.

Light-Chain Filtering Process

Stage 1:

1. North cluster: L1 = L1-16,17-A, L2 = L2-8-A, L3 = L3-9,10-A: 101,166 antibodies remaining;
2. Human germline identity > = 85:74,839 antibodies remaining;
3. Orientation residues (36, 38, 42, 43, 44, 87, 98) match template residues (100% identity): 22,094;
4. Number of rare amino acids in the template (occurring in <1% of human residues in the antibody database) < = 5:21,184.

Stage 2:

5. Total rare amino acids after template CDR porting. Given the template CDRs may have unusual residues, the scaffolds are re-filtered to have less than 5 rare amino acids after porting the template CDRs (note that this looks redundant compared to step 4, but is necessary because the DB only stores total number of rare amino acids);
6. The PKA score is computed as a simple heuristic for antibody charge.

Stage 3:

7. Scaffolds are sorted by their loop homology to the template, rare amino acids, and PKA scores and then sampled such that the final scaffold set will have a hamming distance of at least 12 between all final scaffolds; 45 scaffolds remained in the end.

Stage 4:

8. Scaffolds satisfying the T-cell epitope criterion (no position should have more than the maximum number of alleles flagged at that position among CSTs, and less than 5 positions should have more than the 90th percentile of alleles flagged at that position among CSTs) are considered for use in the candidate designs; 10 scaffolds remained in the end.

### 4.4. CDR–Framework Shuffling

The CDR–framework shuffling consisted of porting the selected CDRs onto the selected frameworks. Given the time and experimental resource constraints, of the 8 VH scaffolds returned from the above filtering procedure, the top 4 (the first 4 selected in the sampling process) were chosen for shuffling. Similarly, 3 VL scaffolds were chosen. The CDRs involved in the shuffling process included the template CDRs and 5 H1 CDRs, 3 H2 CDRs, 6 H3 CDRs, 4 L1 CDRS, 2 L2 CDRS, and 3 L3 CDRs, selected as described above and detailed in Table 1.

After ranking the CDRs and frameworks using the methodology described above, CDR combinations were ported onto frameworks following these rules:

1. At least 1 HCDR and 1 LCDR came from the candidate pool in each construct;
2. All candidate CDRs were used at least once;
3. A given non-template CDR was used in at most 2 VH or VL chains, minimizing the risk of oversampling a deleterious CDR and losing valuable data on CDRs from the other paired chains;
4. Each framework was paired with at least 2 non-template CDRs at least once, to ensure sufficient sampling diversity.

The CDR–framework combinations were generated by iterating through the ranked candidate frameworks and CDRs, ensuring each of the above rules were met.

### 4.5. Expression and Purification of Recombinant Monoclonal Antibodies

The variable heavy- and light-chain sequences of the known anti-SARS CoV-2 antibody H4 and variants were cloned into the full-length IgG1 expression vectors pcDNA3.3 HC and pcDNA3.3 LC (ATUM). The recombinant antibodies were transiently expressed in both ExpiCHO and Expi293 cells according to the manufacturer’s protocol (Invitrogen). The supernatants from 1 mL transient transfections of the antibodies were purified using the AssayMAP BRAVO platform with 5 mL Protein A cartridges (Agilent Technologies). Lager scale transient transfections were purified on the Akta FPLC system using 1 mL of HiTrap MabSelect PrismA™ affinity chromatography resin (Cytiva). The purified recombinant monoclonal antibodies were stored in 1x phosphate-buffered saline at 4 °C until use. Specific site-directed mutations on the H4 antibody sequence were performed using the Quick-Change Site-Directed Mutagenesis Kit II (Agilent technologies).

### 4.6. Screening of Expressed Recombinant Antibodies Using an Enzyme-Linked Immunosorbant Assay (ELISA)

The antibodies purified from a 1 mL transient transfection were tested for their binding against SARS CoV-2 S1 (Sino#40591-V08H) and RBD (Acro#SPD-C52H3) proteins using an ELISA. Briefly, 2 μg/mL of either SARS CoV-2 RBD or S1 protein were coated onto 96-well ELISA plates (Nunc Maxisorp) and left overnight at 4 °C. The wells were blocked with 5% Blotto (Santa Cruz) in 1× PBST for 1 h at room temperature. After rinsing the plates three times, 1:100 and 1:10,000 dilutions of the purified recombinant antibody solution were added to the plates, which were incubated on a rocker platform for 2 h at room temperature. After rinsing the plates three times with 1× PBST, a rabbit anti-human IgG conjugated to horseradish peroxidase (Jackson Immuno Research) was added to each well. The plates were incubated for 1 h at room temperature followed by washing with 1× PBST and the addition of the TMB substrate. The reaction was stopped by adding 1 N of sulfuric acid and the absorbance was read at 405 nm.

### 4.7. Affinity Determination using Octet (Biolayer Interferometry)

The affinity of the antibody to SARS CoV-2 RBD was determined using AHC sensors that were loaded with 0.5 μg/mL of antibody after pre-soaking the sensors in 20 mM of HEPES buffer with 150 mM of NaCl and 0.05% Tween 20 at pH 7.4 or assay buffer. The antibody-coated sensors were blocked in a 20 mM of HEPES buffer with 150 mM of NaCl, 0.05% Tween 20, and 2% BSA for 300 s. A two-fold dilution of the SARS CoV-2 RBD from 125 nM to 3.906 nM in assay buffer was made and the antibody-coated AHC sensors were then incubated in the various dilutions followed by dissociation in assay buffer. The Kd values were calculated using the global fit method on the Octet Red96 (Sartorius) instrument.

### 4.8. ELISA-Based ACE2 Inhibition Assay

The ability of the antibody to block the binding of SARS CoV-2 RBD and ACE2 was determined using the SARS-CoV-2 Inhibitor Screening Kit (ACRO biosystems), which helps in rapid high-throughput screening. In this assay, the CoV-2-RBD is immobilized on the ELISA plate and the constant concentration of biotinylated human ACE2 and dilution series of the antibody compete against each other to bind to CoV-2-RBD. The assay was carried out according to the manufacturer’s protocol.

### 4.9. Generation of SARS-CoV-2 Pseudoviral Particle

The genes of SARS2-CoV-2 Spike 614D were synthesized via codon optimization. The genes were then cloned into the pcDNA3.1 vector. The pseudoviral particle was made using the following procedure. Three plasmids pCMV-MLV-gag-pol, pTG-Luc, and the SARS2-CoV-2 spike expression vector were transfected into 293T cells at a ratio of 3:4:3 using the Lipofectamine 3000 transfection reagent (ThermoFisher). Next, 48 h post-transfection, the supernatants were collected and centrifuged at 290 g for 7 min. The supernatants were then passed through a 0.45 μm syringe filter.

### 4.10. Pseudovirus Neutralization Assay

To prepare for the pseudovirus assay, 7.5 × 10^3^ HEK293-ACE2 cells, which stably express full-length human ACE2, were plated into each well of a 384-well white-clear plate coated with poly-D-Lysine in 15 µL of culture medium. On the 2nd day, the monoclonal antibodies were diluted (5× the final concentration) in the culture medium. Next, 12.5 µL samples of SARS-CoV-2 MLV pseudoviruses were mixed with 5 µL of each monoclonal antibody (5× the final concentration) and incubated at 37 °C for 1 hr. After removing the medium in each well, 17.5 μL of the individual antibody–virus mixture was added. The plate was centrifuged at 54 g for 15 min at 4 °C and 7.5 μL of the culture medium was then added. The total final volume in each well was 25 μL. The cells were then incubated at 37 °C for 42 h. The luciferase activities were measured with a Firefly Luciferase Assay Kit (CA-L165, eEnzyme) according to the protocols in the instructions. The IC50 values were calculated based on the curve fitting in GraphPad Prism. The data were normalized as relative infectivity values.

### 4.11. Plaque Reduction Neutralization Test (PRNT)

The PRNT was performed as described earlier [39]. The SARS-CoV-2 reference virus (Wuhan-Hu-1) strain was used to infect the Vero E6 cells. The 100% infectivity control process was performed using different viral titers [39]. The infectivity was determined using plaque counting based on manual virus plaque formation sighting on the plates. The negative control included blank cells without the virus. The neutralization assay was performed at various dilution concentrations of the antibodies (starting from 100 ug/mL and serially diluted over 8 dilutions), and for each dilution the readouts were obtained in triplicate.

## Data Availability

Not applicable.

## Supplementary Materials

The following supporting information can be downloaded at: https://www.mdpi.com/article/10.3390/v14122694/s1, Figure S1: Immunogenicity profiling of CB79; Figure S2: Antibody screening and selection process that led to CB79; Figure S3: Biophysical characterization of CB79; Figure S4: Analysis of the contributions of CDR mutations to the CB79 antibody-antigen interface; Figure S5: Alignment of amino acid sequence between CB79, scaffold, and H4; Table S1: Binding kinetic constants generated using BLI for the CB79 and H4 point mutants.
