# Supplementary material for: Optimizing Antibody Affinity and Developability Using a Framework–CDR Shuffling Approach—Application to an Anti-SARS-CoV-2 Antibody
Source: Viruses. 2022 Nov 30;14(12):2694. doi: 10.3390/v14122694 (PMC9784564; doi:10.3390/v14122694)
Supplement: Supplementary file 1 [file viruses-14-02694-s001.zip › viruses-2008478-supplementary.pdf]

SUPPLEMENTARY FIGURES

Supplementary Figure S1. Immunogenicity profiling of CB79. Frequency (e.g. number) of canonical alleles with T-cell epitope binding cores at each position on the heavy and light variable regions of PmAb79 is dotted in blue. Positions 95-A,B,C on the light chain are in some CST antibodies and are therefore are displayed, but these positions do not occur in CB79. The CST antibodies considered are human or humanized mAbs in trials or approved as of November 2019. T-Cell epitopes are evaluated using netMHCIIPan3.2 software [28]. The CST Range depicts the maximum canonical allele frequency at each position for all considered CST mAbs. PmAb79 is within the CST range for all positions in both the heavy and the light chains.

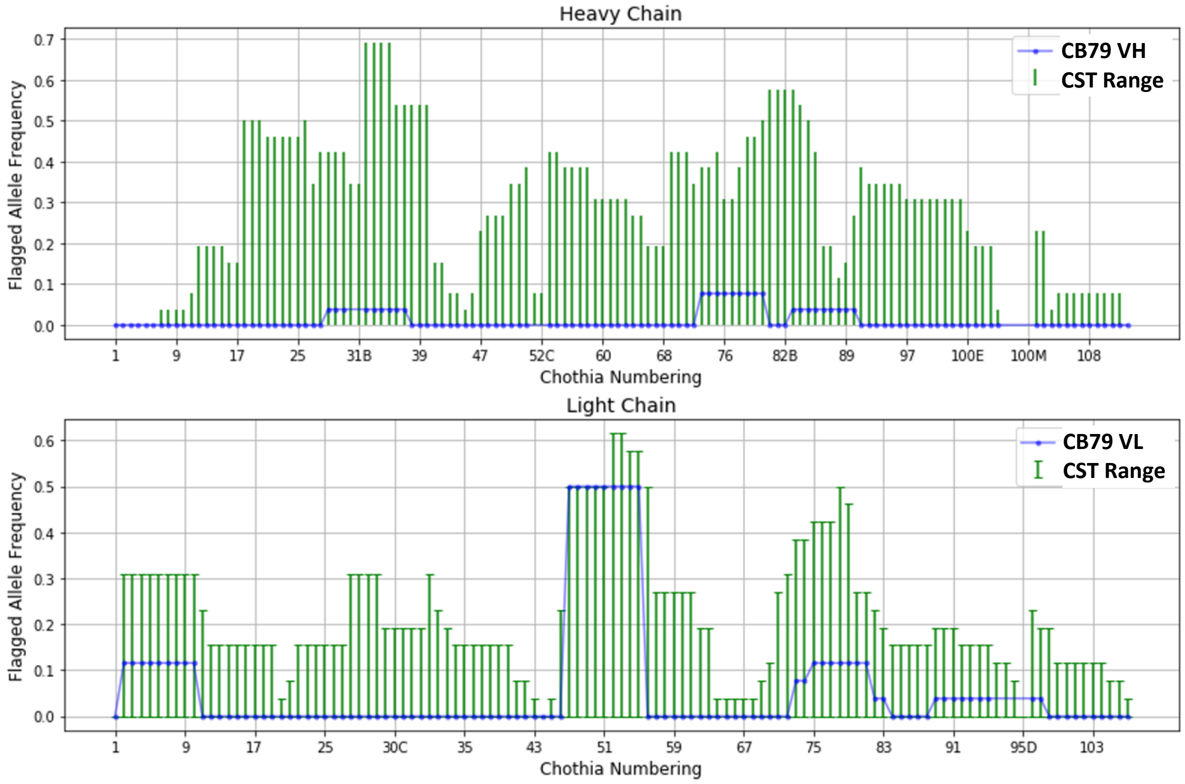

Supplementary Figure S2. Antibody screening and selection process that led to CB79.

A, shows the nomenclature corresponding to the pairing of different heavy and light chains. The frameworks and CDRs of VH and VL (**Tables 1 and 2**) are classified into different groups, which are assigned integer values (0, 1,…). **B**, shows expression levels of the designed antibodies normalized relative to the template antibody H4. **C**, screening antigen-binding property of designed antibodies using ELISA where the OD is normalized to that of the template antibody H4.

A.

				construct name							
			**LF_group**	**0**	**1**			**2**		**3**	
			**L1_group**	**0**	**1**		**4**	**0**	**2**	**0**	**3**
			**L2_group**	**0**	**0**	**1**	**2**	**1**	**0**	**0**	**0**
			**L3_group**	**0**	**0**	**1**	**0**	**0**	**1**	**2**	**3**
**HF_group**	**H1_group**	**H2_group**	**H3_group**								
**0**	**0**	**0**	**0**	R0							
**1**	**1**	**0**	**0**		CB1	CB3	CB2	CB4	CB5	CB6	CB7
		**1**	**2**		CB15	CB17	CB16	CB18	CB19	CB20	CB21
	**2**	**0**	**1**		CB8	CB10	CB9	CB11	CB12	CB13	CB14
**2**	**0**	**0**	**2**		CB22	CB24	CB23	CB25	CB26	CB27	CB28
	**3**	**0**	**3**		CB29	CB31	CB30	CB32	CB33	CB34	CB35
		**2**	**1**		CB36	CB38	CB37	CB39	CB40	CB41	CB42
**3**	**0**	**1**	**0**		CB43	CB45	CB44	CB46	CB47	CB48	CB49
		**2**	**4**		CB50	CB52	CB51	CB53	CB54	CB55	CB56
	**2**	**3**	**3**		CB57	CB59	CB58	CB60	CB61	CB62	CB63
**4**	**0**	**3**	**0**		CB64	CB66	CB65	CB67	CB68	CB69	CB70
	**4**	**0**	**5**		CB71	CB73	CB72	CB74	CB75	CB76	CB77
	**5**	**0**	**6**		CB78	CB80	CB79	CB81	CB82	CB83	CB84

B.

				expression score							
			**LF_group**	**0**	**1**			**2**		**3**	
			**L1_group**	**0**	**1**		**4**	**0**	**2**	**0**	**3**
			**L2_group**	**0**	**0**	**1**	**2**	**1**	**0**	**0**	**0**
			**L3_group**	**0**	**0**	**1**	**0**	**0**	**1**	**2**	**3**
**HF_group**	**H1_group**	**H2_group**	**H3_group**								
**0**	**0**	**0**	**0**	1.00							
**1**	**1**	**0**	**0**		1.03	0.97	0.48	0.41	0.78	0.96	0.48
		**1**	**2**		0.98	0.62	1.01	0.92	0.21	0.89	0.36
	**2**	**0**	**1**		0.98	0.90	0.43	0.96	0.20	0.86	0.48
**2**	**0**	**0**	**2**		0.57	0.64	1.09	0.88	-0.01	0.84	0.86
	**3**	**0**	**3**		0.54	0.48	1.03	0.81	0.56	0.47	0.78
		**2**	**1**		0.38	0.95	0a.87	0.31	0.68	0.43	0.89
**3**	**0**	**1**	**0**		0.95	0.96	0.64	0.29	0.12	0.78	0.76
		**2**	**4**		0.98	0.99	0.65	0.10	0.80	0.36	0.69
	**2**	**3**	**3**		0.95	0.86	0.48	0.68	0.50	0.90	0.40
**4**	**0**	**3**	**0**		0.87	1.01	1.10	0.81	0.04	0.85	0.37
	**4**	**0**	**5**		0.54	0.54	1.10	0.87	0.11	0.84	0.33
	**5**	**0**	**6**		1.20	0.53	0.99	0.81	0.02	0.79	0.94

C.

				Spot ELISA Absorbance							
			**LF_group**	**0**	**1**			**2**		**3**	
			**L1_group**	**0**	**1**		**4**	**0**	**2**	**0**	**3**
			**L2_group**	**0**	**0**	**1**	**2**	**1**	**0**	**0**	**0**
			**L3_group**	**0**	**0**	**1**	**0**	**0**	**1**	**2**	**3**
**HF_group**	**H1_group**	**H2_group**	**H3_group**								
**0**	**0**	**0**	**0**	1.00							
**1**	**1**	**0**	**0**		1.24	0.05	0.98	1.08	0.07	0.97	0.21
		**1**	**2**		0.04	0.03	0.03	0.03	0.04	0.06	0.03
	**2**	**0**	**1**		0.07	0.03	0.04	0.05	0.03	0.05	0.03
**2**	**0**	**0**	**2**		0.04	0.04	0.03	0.05	0.06	0.04	0.03
	**3**	**0**	**3**		0.05	0.03	0.04	0.04	0.04	0.04	0.04
		**2**	**1**		0.04	0.04	0.03	0.03	0.04	0.05	0.04
**3**	**0**	**1**	**0**		0.04	0.04	0.03	0.04	0.03	0.05	0.04
		**2**	**4**		0.04	0.03	0.04	0.04	0.04	0.04	0.04
	**2**	**3**	**3**		0.04	0.03	0.04	0.03	0.03	0.03	0.04
**4**	**0**	**3**	**0**		0.03	0.03	0.03	0.03	0.03	0.04	0.04
	**4**	**0**	**5**		1.27	0.04	0.90	0.92	0.06	1.10	0.24
	**5**	**0**	**6**		1.63	0.40	1.34	1.28	0.83	1.13	0.65

Supplementary Figure S3. Biophysical characterization of CB79: Percent monomer content, (viz., Purity level) determined using SEC-HPLC (A) and thermal stability determined using DSC (B).

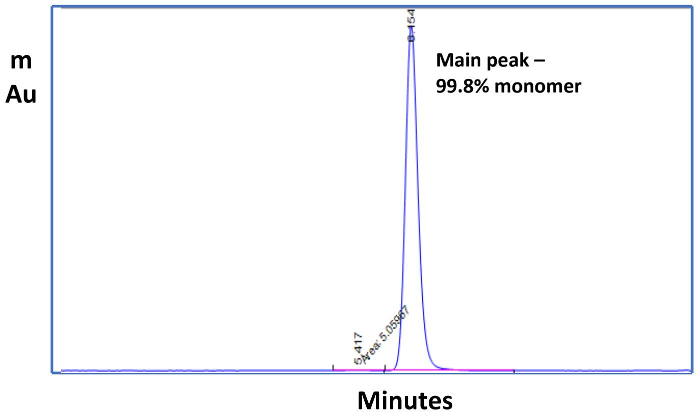

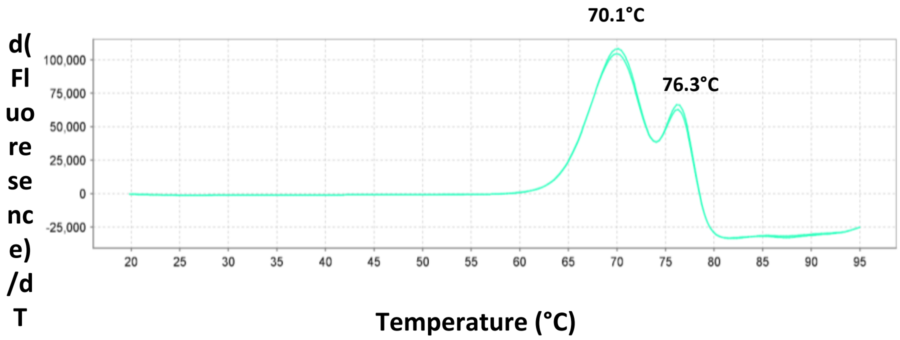

Supplementary Figure S4. Analysis of the contributions of the CDR mutations to the CB79 antibody-antigen interface. Briefly, we developed the CB79 complex based on the H4-spike complex (PDB: 7L58) using PyRosetta [37] (CBMID: 20061306), modelled the missing atoms and relaxed the structure using force field energy minimization.

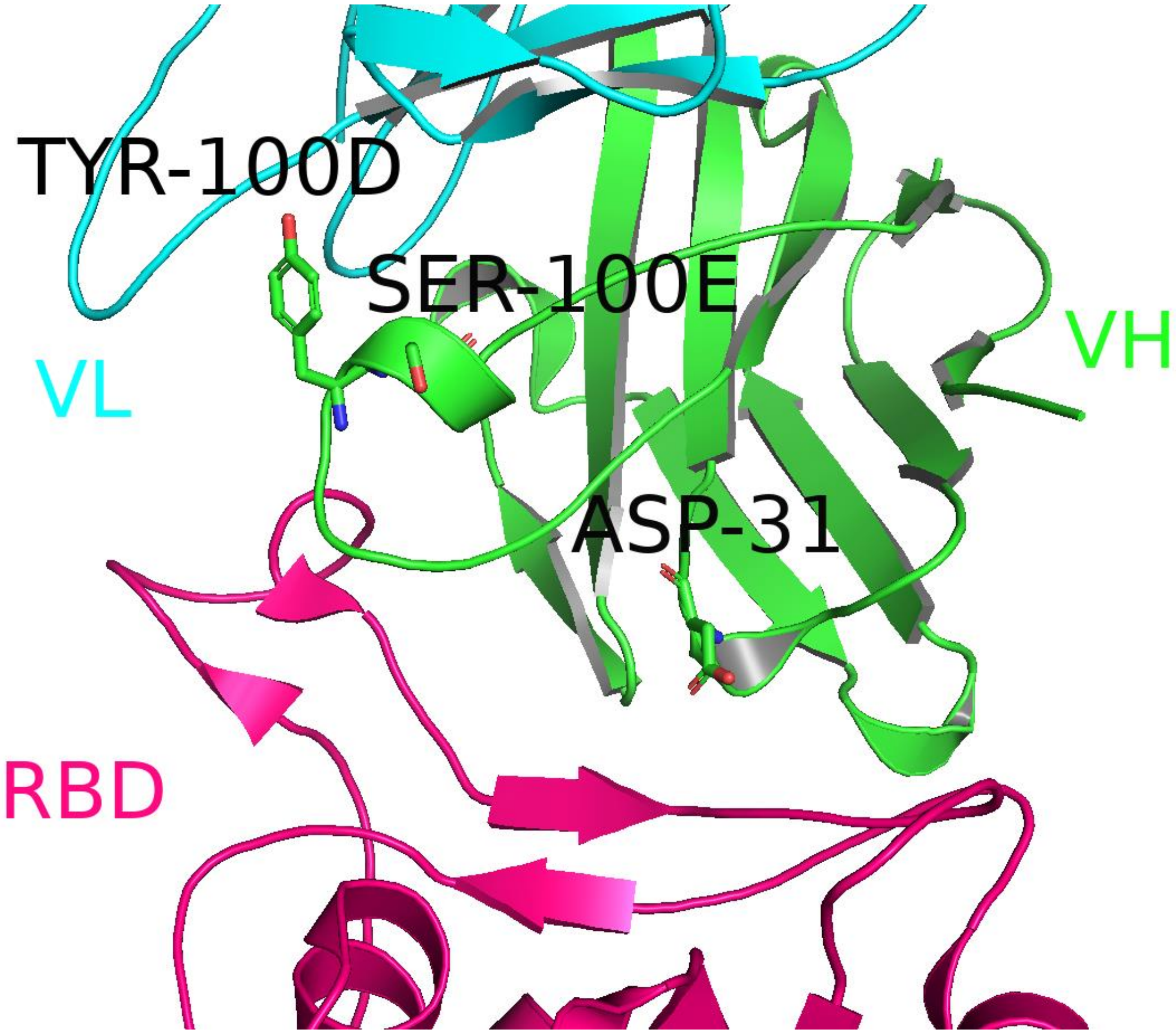

Supplementary Figure S5. Alignment of VH-VL amino acid sequence between CB79, scaffold and H4. Residues are colored by their Position in the 3D structure: blue: epitope-paratope interface; red: CDR-FWR interface; green: developability based mutations; purple: VH/VL interface.

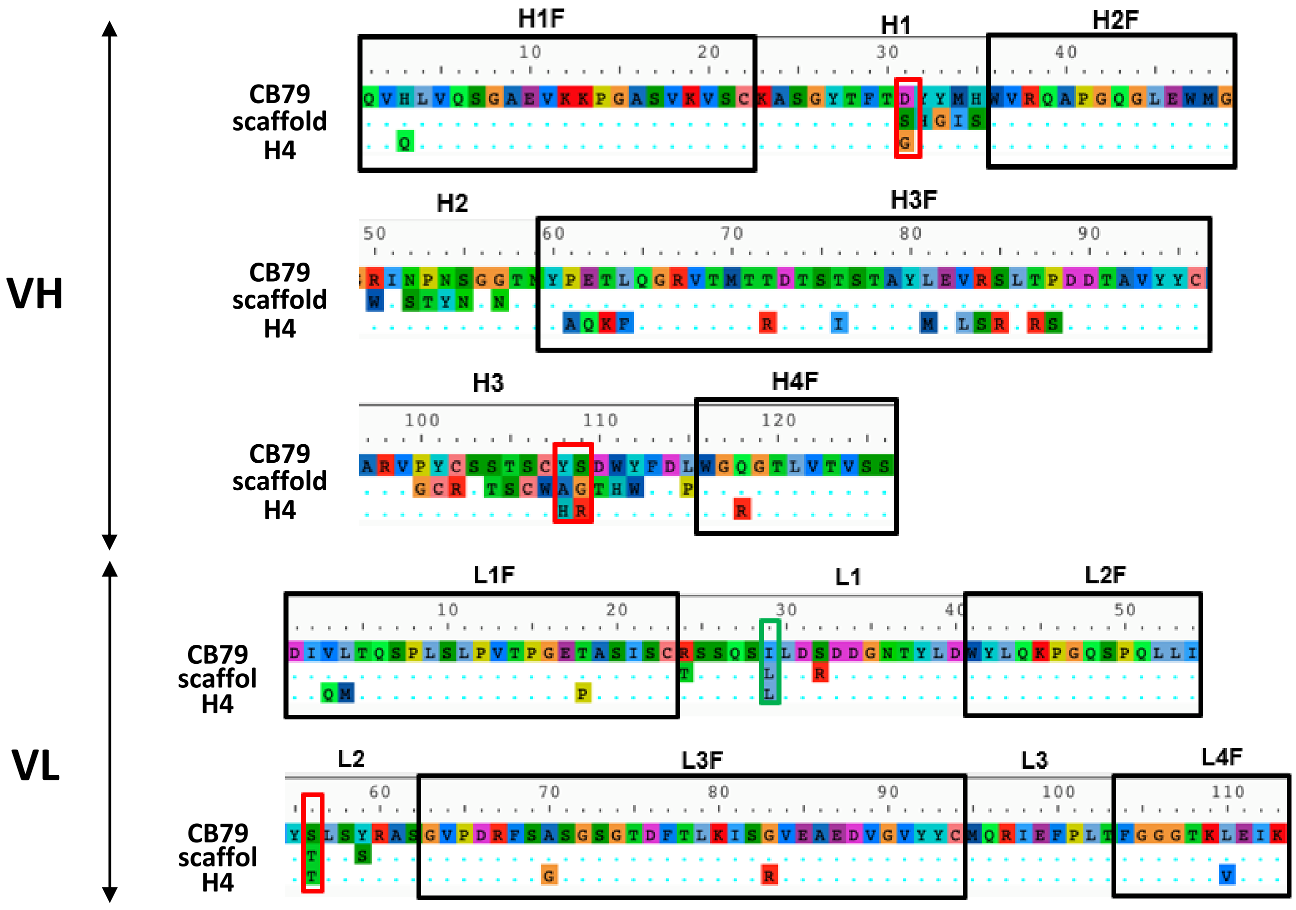

Supplementary Table S1. Binding kinetic constants generated using BLI for the H4 and CB79 point mutants.

Location	Mutation introduced on H4 (H4*)	KD (H4) / KD (H4*)	Reverse mutation introduced on CB79 (CB79*)	KD (CB79)) / KD (CB79*)
HCDR1	G31->D	1.4	D31->G	1.8
HCDR3	H100D->Y	2.2	Y100D->H	.35
HCDR3	R100E->S	1.9	S100E->R	2.1
